# Diabetes mellitus was not associated with lower amputation-free survival after open revascularization for chronic limb-threatening ischemia – A nationwide propensity score adjusted analysis

**DOI:** 10.1177/1358863X211008249

**Published:** 2021-05-18

**Authors:** Erika Lilja, Anders Gottsäter, Mervete Miftaraj, Jan Ekelund, Björn Eliasson, Ann-Marie Svensson, Moncef Zarrouk, Stefan Acosta

**Affiliations:** 1 Department of Clinical Sciences, Lund University, Malmö, Sweden; 2 Department of Cardio-Thoracic Surgery and Vascular Diseases, Vascular Center, Skåne University Hospital, Malmö, Sweden; 3 Centre of Registers, National Diabetes Register, Göteborg, Sweden; 4 Department of Molecular and Clinical Medicine, Institute of Medicine, University of Gothenburg, Göteborg, Sweden

**Keywords:** amputation, bypass, chronic limb-threatening ischemia (CLTI), diabetes mellitus

## Abstract

The risk of major amputation is higher after urgently planned endovascular therapy for chronic limb-threatening ischemia (CLTI) in patients with diabetes mellitus (DM). The aim of this nationwide cohort study was to compare outcomes between patients with and without DM following urgently planned open revascularization for CLTI from 2010 to 2014. Out of 1537 individuals registered in the Swedish Vascular Registry, 569 were registered in the National Diabetes Register. A propensity score adjusted Cox regression analysis was conducted to compare outcome between the groups with and without DM. Median follow-up was 4.3 years and 4.5 years for patients with and without DM, respectively. Patients with DM more often had foot ulcers (*p* = 0.034) and had undergone more previous amputations (*p* = 0.001) at baseline. No differences in mortality, cardiovascular death, major adverse cardiovascular events (MACE), or major amputation were observed between groups. The incidence rate of stroke was 70% higher (95% CI: 1.11–2.59; *p* = 0.0137) and the incidence rate of acute myocardial infarction (AMI) 39% higher (95% CI: 1.00–1.92; *p* = 0.0472) among patients with DM in comparison to those without. Open vascular surgery remains a first-line option for a substantial number of patients with CLTI, especially for limb salvage in patients with DM. The higher incidence rates of stroke and AMI among patients with DM following open vascular surgery for infrainguinal CLTI require specific consideration preoperatively with the aim of optimizing medical treatment to improve cardiovascular outcome postoperatively.

## Introduction

Chronic limb-threatening ischemia (CLTI) is the end-stage of peripheral artery disease (PAD) and should be viewed as a sign of systemic atherosclerosis with a high mortality in stroke and myocardial infarction.^1^ The 1-year mortality rate in CLTI patients is predicted to be 22–26%.^2,3^ Smoking and diabetes mellitus (DM) are the strongest risk factors for PAD,^4^ with an expected increase of the DM incidence in the United States by 200% from 2005 to 2050.^5^

Individuals with both DM and PAD have a more distal distribution of the arterial disease, and tend to have more significant comorbidities.^6^ In view of these factors, minimal invasive endovascular therapy for CLTI among patients with DM might induce less myocardial stress^7^ compared to open surgery, and therefore be more beneficial to achieve higher amputation-free survival.

Indeed, endovascular therapy was associated with a lower risk of mortality in patients with type 2 diabetes compared to those without DM among patients with infrainguinal CLTI.^8^ However, endovascular therapy was also associated with a higher risk of major amputation in patients treated with insulin only compared to those without DM.^8^ In a recent nationwide propensity adjusted analysis, patients with DM undergoing endovascular therapy had lower amputation-free survival and a clearly higher risk of major amputation compared to those without DM.^9^ Since the proportion of patients with DM and CLTI undergoing elective endovascular therapy is increasing compared to open vascular surgery,^10–12^ it is of great importance to investigate whether or not the results after urgently planned open vascular surgery are also associated with similar inferior results in DM patients.

The main aim of this study was to evaluate the risk of major amputation and mortality after urgently planned open vascular surgery in patients with CLTI and infrainguinal arterial disease, comparing patients with DM and without DM in a nationwide propensity score adjusted analysis.

## Methods

The present cohort study was based on prospectively collected data of all Swedish patients with CLTI undergoing urgently planned open vascular surgery for infrainguinal arterial disease between 2010 and 2014, in total 1537 patients. CLTI was defined as the presence of PAD along with rest pain, gangrene, or ulcers for > 2 weeks.^1^ Individuals without tissue loss (ulcer or gangrene) were regarded to have rest pain.

### Databases and procedures

By using the personal identity number, unique to every Swedish citizen, information on individual patient data was obtained from nationwide population-based databases. Subjects were identified by cross-matching the Swedish Vascular Register (Swedvasc)^13^ and the Swedish National Diabetes Register (NDR).^14^ Duplicated patients were excluded.

Data were also retrieved from several national registries including the National Patient Register (NPR) and the Longitudinal Integration Database for Health Insurance and Labour Market Studies which was used for information of socioeconomic characteristics.^15,16^ Country of birth and level of education was retrieved and stratified in to three groups – compulsory school, upper secondary school, and college or university – and marital status as married, separated, single, or widowed.

Information of time and cause of death were retrieved from the Swedish Cause of Death Register.^17^ Information about comorbidities and drug treatment at baseline was retrieved from the Prescribed Drug Register (PDR)^18^ and the cancer registry.^19^ The NPR provides information on discharge diagnoses and length of hospital stay since 1987 with > 99% coverage, with a positive predictive value of > 99% for vascular interventions for lower limb ischemia.^15^

All patients undergoing vascular surgery in Sweden are registered in Swedvasc. Pre- and perioperative data regarding type of treatment (acute or elective, endovascular or open), risk factors, complications, and reinterventions are recorded. Patient follow-up is at 30 days and 12 months following the surgical procedure. Only the first open vascular procedure within the study period was analyzed, regardless of whether it was a repeat or first procedure. Repeated open vascular surgery in the ipsilateral or contralateral limb during the study period was not assessed.

The NDR was, in 2019, estimated to cover 88% of Swedish citizens over the age of 18 with DM.^20^ It contains data on clinical characteristics, diabetes treatment, risk factors, and diabetic complications. Each individual gave consent to inclusion in the register. As Swedvasc provides only 1-year follow-up after vascular surgery, the Swedish NPR was used to gain further information on outcomes, comorbidities, and discharge diagnoses.

**Figure 1.**
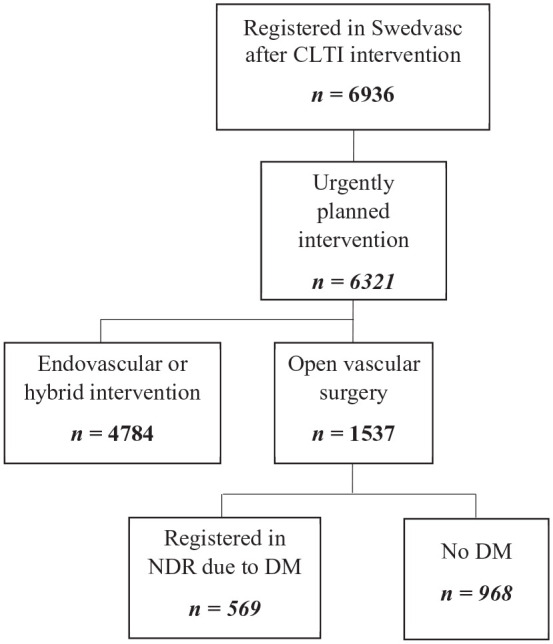
Flow chart of patients in the Swedish Vascular Register (Swedvasc) undergoing urgently planned open vascular surgery for CLTI during 2010 to 2014. Further division was done according to whether the patient was registered in NDR due to DM or not. CLTI, chronic limb-threatening ischemia; DM, diabetes mellitus; NDR, National Diabetes Register.

In this observational case–control study, patients registered in the Swedvasc infrainguinal module from 2010 to 2014 due to urgently planned open vascular surgery were identified. Patients with a corresponding registration in NDR between 2009 and 2015, thus having DM, were compared to those without such registration (not having DM) (Figure 1).

### Baseline data

The NPR uses the *International Classification of Diseases, Tenth Revision* (ICD-10) for classification of diagnoses. Comorbidities at baseline included: atrial fibrillation or flutter, heart failure, coronary heart disease, hypertension, and stroke. Furthermore, renal disorder (kidney transplant, renal failure, or dialysis), cancer, liver disease, psychiatric disorders (excluding dementia), and chronic obstructive pulmonary disease were included. CLTI-related variables such as previous amputation, tissue loss, thromboendarterectomy, and previous bypass surgery were also included. The bypass variable was divided into two groups: vein bypass or synthetic bypass. Acute myocardial infarction (AMI) was defined as I21 (ICD-10). Renal impairment was defined as an estimated glomerular filtration rate (eGFR) < 60 mL/min/1.73 m^2^ with data from NDR on individuals with DM only.

Smoking was defined as current smoking at baseline and the information was retrieved from Swedvasc. When smoking data were missing in Swedvasc, NDR data were used to complement. Drug treatment was defined according to the PDR. Use of lipid-lowering drugs, acetylsalicylic acid (ASA), metformin and other glucose-lowering medications, and anticoagulant therapy was included. Hypertension was defined as collecting a minimum of one prescription of antihypertensive drugs 1 year prior to the index operation. Three months of medicine use is equivalent to one prescription. Use of ASA and lipid-lowering medication was defined similarly.

Amputation was defined as amputation above the ankle (e.g., major amputation). Since the NPR is a code-based register, information on amputation laterality was not always possible to determine. A recent validation of major amputation for CLTI has been performed by reviewing 1366 patients’ medical records, showing < 10% missing data for amputation with remaining uncertainty of the laterality of the amputation.^21^ MACE was defined as angina pectoris, acute myocardial infarction, ischemic heart disease, stroke and intracranial hemorrhage.

### Follow-up

Follow-up started the date the patients were revascularized, defining the index date, and continued up to December 31, 2016 for endpoints using Swedvasc and until December 31, 2017 for mortality. This was enabled through linkage between NDR and the Cause of Death Register with causes and time of death.

### Statistical analysis

Outcomes were compared after urgently planned open vascular surgery for CLTI between patients with and without DM by propensity score adjusted analysis. A propensity score technique to adjust for multiple risk factors^22,23^ was used since multivariate adjustments by logistic regression is limited by the number of endpoints, and a limited number of covariates should be modelled.^24^ The propensity scores were estimated using a generalized boosted multinomial regression model with an interaction depth of 3, a maximum of 75,000 trees, and a shrinkage of 0.01. The optimal number of trees was selected using a stopping rule applied to the degree of balance.

The distribution of propensity scores varies between infrainguinal CLTI patients with and without DM, requiring some form of adjustment for confounding. To avoid losing patients in a matching procedure inverse probability of treatment (here defined as having DM), weighting (IPTW) was chosen. It should be noted that baseline diabetes treatment was excluded from the estimation of the weights and therefore not adjusted for.

Descriptive statistics were presented using mean, SD, counts, and percentages according to variable type. The degree of similarity between infrainguinal CLTI patients with and without DM was described using the standardized mean difference (SMD) and *p*-values. Cumulative mortality and major amputation were described using Kaplan–Meier curves transformed to estimate the distribution function rather than the survival function.

The effects of diabetes duration, HbA1c, renal impairment, and tissue loss in the group with DM were evaluated by fitting a Cox proportional hazards model. The model included gender, age, diabetes duration, HbA1c, renal impairment, and tissue loss at baseline. Only patients with nonmissing values on gender, age, diabetes duration, HbA1c, renal impairment, and tissue loss were included in the analysis.

### Sensitivity analysis of the inverse probability of treatment weighting adjusted analysis

The IPTW adjusted analysis was performed using all patients. Sensitivity analyses were performed by placing a threshold on the weight (e.g., maximum weight = 10) and by trimming the data set based on the value of the propensity score (e.g., keeping datapoints above the 2.5% and 1% percentile determined for the DM + CLTI group and below the 97.5% and 99% percentile determined for the CLTI group).

Results when truncating maximum weight at 10 were very similar to the main results, indicating that there is no large influence by larger weights on the analysis. Results when trimming data based on percentiles of the propensity scores were largely consistent with the main analysis.

The statistical analyses compared CLTI patients with DM to CLTI patients without DM using both an unadjusted and an IPTW adjusted Cox regression model. IPTW adjusted Cox regression analysis was expressed as hazard ratios (HR) with 95% CI. See the online supplementary material (Appendix 1) for a list of the adjusted variables. Analyses were performed using R 3.4.3 (http://cran.us.r-project.org/). A *p* < 0.05 was considered statistically significant.

### Ethical approval

The study was approved by the regional research ethical committee in Lund, Sweden (2016/232 and 2016/544). As all patients had consented to being reported in NDR and Swedvasc, no individual consent was required to be included in this study according to Swedish law.

## Results

### Study population and demographic characteristics

Between 2010 and 2014, a total of 1537 individuals underwent urgently planned open vascular surgery for CLTI, of whom 569 had DM and were registered in Swedvasc (Figure 1). Median follow-up was 4.3 years (IQR 2.2–5.7) and 4.5 years (IQR 2.5–5.9) for patients with and without DM, respectively. Table 1 presents unadjusted baseline data along with clinical and demographic characteristics for the two groups. The majority of patients with DM (88.6%) were classified as type 2, 9.8% as type 1, and 1.6% as having other or unspecified types of DM. Among individuals with DM, 20.9% were not treated with any glucose-lowering agents.

**Table 1.** Baseline characteristics of patients with CLTI, with and without DM, undergoing urgently planned open vascular surgery.

	DM and CLTI	CLTI	*p*-value	SMD
	n = 569	n = 968		
Age, years, mean (SD)	73 (9.29)	76 (9.10)	< 0.001	0.307
Women, *n* (%)	217 (38.1)	512 (52.9)	< 0.001	0.300
Smoking, *n* (%)	113 (22.1)	238 (30.1)	0.002	0.182
Duration of DM, years (IQR)	14 (15.75)	–		
HbA1c, mmol/mol (IQR)	57 (18)	–		
Medication, *n* (%)				
Lipid-lowering	468 (82.2)	713 (73.7)	< 0.001	0.208
Metformin	228 (40.1)	–		
Glucose-lowering agents	450 (79.1)	–		
Acetylsalicylic acid	435 (76.4)	760 (78.5)	0.381	0.049
Clopidogrel	115 (20.2)	120 (12.4)	< 0.001	0.213
Anticoagulant therapy	242 (42.5)	334 (34.5)	0.002	0.166
Antihypertensive	546 (96.0)	814 (84.1)	< 0.001	0.404
ACE inhibitor	305 (53.6)	348 (36.0)	< 0.001	0.361
ARB	199 (35.0)	207 (21.4)	< 0.001	0.306
Alpha blocker	23 (4.0)	15 (1.5)	0.004	0.152
Beta blocker	365 (64.1)	496 (51.2)	< 0.001	0.264
Calcium channel blocker	258 (45.3)	345 (35.6)	< 0.001	0.199
Diuretic	330 (58.0)	468 (48.3)	< 0.001	0.194
Digoxin	38 (6.7)	38 (3.9)	0.023	0.123
Nitrate	134 (23.6)	176 (18.2)	0.014	0.132
Income, quartile (%)			0.037	0.156
1	107 (18.8)	243 (25.1)		
2	141 (24.8)	216 (22.3)		
3	155 (27.2)	256 (26.4)		
4	166 (29.2)	253 (26.1)		
Income * 100 SEK/year, mean (SD)	1,835.59 (2,939.04)	1,663.27 (1,213.73)	0.108	0.077
Education, *n* (%)			0.003	0.184
Compulsory school	310 (55.2)	460 (48.2)		
Upper secondary	203 (36.1)	363 (38.0)		
College or university	49 (8.7)	132 (13.8)		
Civil status, *n* (%)			0.007	0.186
Married	250 (43.9)	374 (38.6)		
Separated	135 (23.7)	203 (21.0)		
Single	56 (9.8)	96 (9.9)		
Widowed	128 (22.5)	295 (30.5)		
Origin, *n* (%)			0.193	0.094
Sweden	478 (84.0)	843 (87.1)		
Europe except Sweden	46 (8.1)	69 (7.1)		
Rest of the world	45 (7.9)	56 (5.8)		
Previous diseases, *n* (%)				
AMI	132 (23.2)	175 (18.1)	0.018	0.127
Coronary heart disease	282 (49.6)	337 (34.8)	< 0.001	0.302
Stroke	86 (15.1)	131 (13.5)	0.433	0.045
Atrial fibrillation	131 (23.0)	197 (20.4)	0.242	0.065
Heart failure	143 (25.1)	173 (17.9)	0.001	0.177
Renal disorder	74 (13.0)	67 (6.9)	< 0.001	0.204
Cancer disease	49 (8.6)	141 (14.6)	0.001	0.187
Liver disease	6 (1.1)	10 (1.0)	1.000	0.002
Psychiatric disorder	19 (3.3)	35 (3.6)	0.888	0.015
COPD	72 (12.7)	159 (16.4)	0.054	0.107
Renal impairment	123 (36.8)	–	–	
Amputation, minor and major	42 (7.4)	33 (3.4)	0.001	0.177
Tissue loss and surgical procedures, n (%)				
Tissue loss	316 (68.1)	518 (62.0)	0.034	0.128
Thromboendarterectomy	179 (31.5)	336 (34.7)	0.19	
Bypass, *n* (%)				
Synthetic or synthetic plus vein bypass	75 (13.2)	165 (17.0)	0.044	
Vein bypass	200 (35.1)	351 (36.3)	0.66	

Categorical variables are presented as number (%) and continuous variables are presented as mean (SD).

Anticoagulant therapy includes vitamin K-antagonists, heparin, low-molecular heparin, DOACs, fondaparinux. Renal impairment was defined as an eGFR <60 mL/min/1.73 m^2^ with data from the Swedish National Diabetes Register. Renal disorder comprises kidney transplant, renal failure or dialysis. Glucose-lowering agents include insulin, oral hypoglycemic agents, and GLP-1 analogues.

ACE, angiotensin converting enzyme; AMI, acute myocardial infarction; ARB, angiotensin II receptor blocker;

CLTI, chronic limb-threatening ischemia; COPD, chronic obstructive pulmonary disease; DM, diabetes mellitus; DOACs, direct oral anticoagulants; eGFR, estimated glomerular filtration rate; GLP-1, glucagon-like peptide-1; HbA1c, hemoglobin A1c; SMD, standardized mean difference.

### Outcome analysis

**Figure 2.**
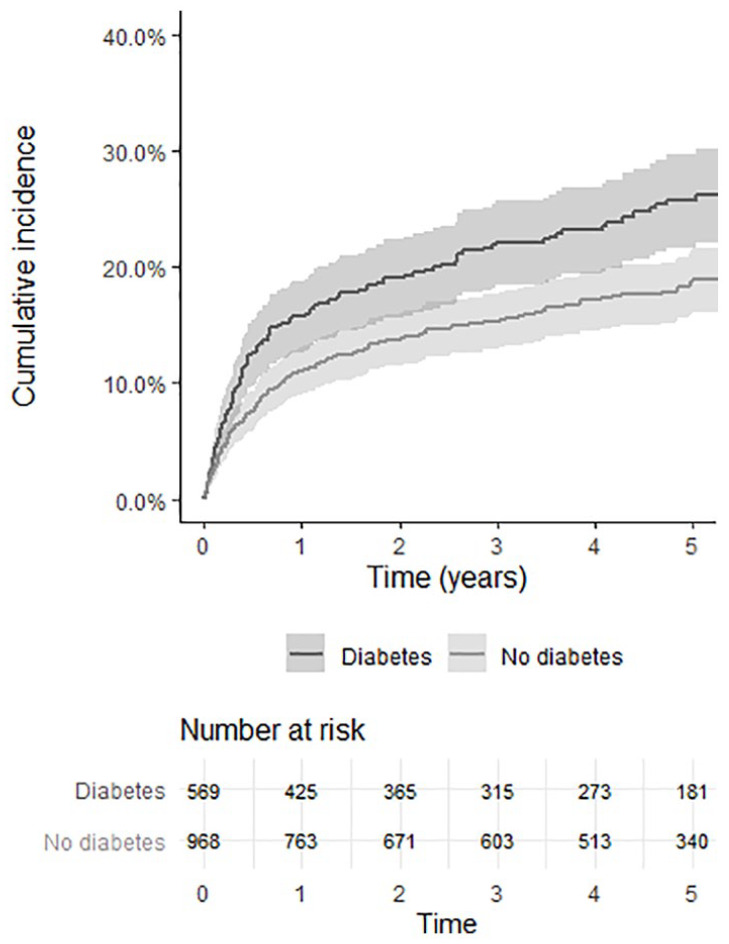
Crude Kaplan–Meier curves showing cumulative incidence of major amputation and total mortality after urgently planned open vascular surgery for CLTI among patients with and without DM. Shaded areas represent standard errors. CLTI, chronic limb-threatening ischemia; DM, diabetes mellitus.

The crude Kaplan–Meier curves for cumulative incidences of major amputation and mortality are displayed in Figure 2. The incidence rate of stroke was 70% higher (95% CI: 1.11–2.59; *p* = 0.0137) and the incidence rate of AMI 39% higher (95% CI: 1.00–1.92; *p* = 0.0472) among patients with DM compared to those without DM. There was no difference in mortality, cardiovascular death, major adverse cardiovascular events (MACE) or major amputation between patients with and without DM (Table 2).

**Table 2.** IPTW adjusted Cox regression analysis of hazard ratio for different endpoints for patients with DM compared to patients without DM after urgently planned open vascular surgery for CLTI.

Endpoint	Hazard ratio	*p*-value	95% CI
Mortality	1.10	0.2504	0.93–1.30
Cardiovascular mortality	1.09	0.4026	0.89–1.33
MACE	1.15	0.0904	0.98–1.34
AMI	1.39	0.0472	1.00–1.92
Stroke	1.70	0.0137	1.11–2.59
Major amputation	1.28	0.0701	0.98–1.66
Major amputation or death	1.15	0.0903	0.98–1.35

AMI, acute myocardial infarction; CLTI, chronic limb-threatening ischemia; DM, diabetes mellitus; IPTW, inverse probability treatment weighting; MACE, major adverse cardiovascular event.

### Effect of diabetes duration, HbA1c, renal impairment, and tissue loss on outcome among patients with diabetes mellitus

Median diabetes duration was 14 years (IQR 7.25–23; *n* = 354), median HbA1c 57 mmol/mol (IQR 49–67; *n* = 366), and median eGFR was 70 mL/min/1.73 m^2^ (IQR 54–91; *n* = 334). Tissue loss was associated with a higher risk of major amputation (HR 2.52, 95% CI: 1.26–5.05; *p* = 0.009) (Table 3). Renal impairment was associated with a higher risk of total mortality (HR 2.13, 95% CI: 1.47–3.08;*p* < 0.001), CV mortality (HR 1.93, 95% CI: 1.26–2.98;*p* = 0.003), and MACE (HR 1.74, 95% CI: 1.25–2.43; *p* = 0.001). Diabetes duration was associated with a higher risk of MACE (HR 1.01, 95% CI: 1.00–1.03; *p* = 0.03).

**Table 3.** Effect of diabetes duration, HbA1c, tissue loss, and renal impairment on different endpoints among patients with DM undergoing urgently planned open vascular surgery for CLTI.

Outcome	Covariate	Hazard ratio	*p*-value	95% CI
Total mortality	Diabetes duration	1.00	0.695	0.98–1.01
	HbA1c	1.01	0.229	0.99–1.02
	Tissue loss	1.35	0.128	0.92–1.99
	Renal impairment	2.13	< 0.001	1.47–3.08
	Diabetes duration	1.00	0.818	0.98–1.02
CV mortality	HbA1c	1.00	0.812	0.99–1.02
	Tissue loss	1.46	0.118	0.91–2.36
	Renal impairment	1.93	0.003	1.26–2.98
MACE	Diabetes duration	1.01	0.030	1.00–1.03
	HbA1c	1.00	0.749	0.99–1.01
	Tissue loss	0.97	0.864	0.69–1.37
	Renal impairment	1.74	0.001	1.25–2.43
AMI	Diabetes duration	1.01	0.395	0.99–1.04
	HbA1c	0.98	0.103	0.95–1.00
	Tissue loss	1.05	0.899	0.52–2.10
	Renal impairment	1.66	0.164	0.81–3.37
Stroke	Diabetes duration	0.98	0.175	0.94–1.01
	HbA1c	0.99	0.597	0.97–1.02
	Tissue loss	1.08	0.829	0.52–2.25
	Renal impairment	1.42	0.339	0.69–2.93
Major amputation	Diabetes duration	1.01	0.512	0.99–1.03
	HbA1c	1.02	0.058	1.00–1.04
	Tissue loss	2.52	0.009	1.26–5.05
	Renal impairment	1.21	0.512	0.68–2.15

The effect of diabetes duration, HbA1c, tissue loss, and renal impairment was evaluated by fitting a Cox proportional hazards model. The model includes gender, age, diabetes duration, HbA1c, tissue loss, and renal impairment at baseline. Only patients with nonmissing values on gender, age, diabetes duration, HbA1c, tissue loss, and renal impairment were included.

Renal impairment was defined as an eGFR < 60 mL/min/1.73 m^2^.

AMI, acute myocardial infarction; CLTI, chronic limb-threatening ischemia; CV mortality, cardiovascular mortality; DM, diabetes mellitus; eGFR, estimated glomerular filtration rate; HbA1c, hemoglobin A1c; MACE, major adverse cardiovascular event.

## Discussion

The present study found a higher incidence rate of stroke and AMI among patients with DM after urgently planned open vascular surgery for infrainguinal CLTI compared to those without DM, whereas there was no difference in mortality, cardiovascular death, MACE or major amputation between patients with DM and without DM.

In the present nationwide study, no difference in major amputation rate following open vascular surgery was found in the group with DM compared to those without DM, despite a higher rate of previous minor and major amputation and tissue loss at baseline in patients with DM. Of note, most patients underwent infrainguinal bypass procedure with vein conduit^25^ without differences between the two groups, which may have contributed to similar results in major amputation. This result differs from our previous study on patients endovascularly revascularized for CLTI, in which patients with DM and CLTI had a higher risk of major amputation.^9^ After bypass surgery for CLTI on the other hand, two previous studies reported no difference in major amputation rate among patients with DM compared to those without DM, despite more advanced occlusive atherosclerotic lesions in DM resulting in a need of a lower level of the distal bypass anastomoses.^26,27^ To be able to achieve equal results in patients with and without DM after bypass, however, it appears necessary to use the saphena magna vein as a bypass conduit, either as reversed bypass^27^ or with an in situ technique,^26^ and that the bypass is performed by a limited number of experienced vascular surgeons.^27^ In a cohort in which approximately 40% had DM, the randomized controlled trial (RCT) BASIL-1 indicated a higher amputation-free survival at 2 years after bypass surgery compared to endovascular therapy for CLTI.^28^ Furthermore, Darling et al. found lower reintervention and restenosis rates following open vascular surgery compared to endovascular therapy among individuals with insulin-dependent DM.^8^ Even though the vascular surgical field in recent years has gone through a major change towards an increased use of endovascular procedures,^11^ open vascular surgery is still the first-line option in a substantial number of patients with CLTI, especially for limb salvage in patients with DM.

In the present study, no difference in mortality was demonstrated, in contrast to data from Swedvasc 2001–2003 where an increased mortality was seen among patients with diabetes after bypass surgery for CLTI.^29^ The results from 2001 to 2003 might partly be explained by a less aggressive use of statins and antiplatelet agents in the past.^30^ The present study showed a higher cumulative incidence rate of stroke and AMI in the group with DM, whereas Swedish patients with CLTI and DM undergoing endovascular therapy had a higher cumulative incidence rate of AMI only.^9^ It is well-known that DM patients have a twofold increased risk of atherothrombotic ischemic stroke compared to those without DM,^31^ and it can be speculated that patients needing an open vascular procedure have a more advanced generalized atherosclerotic disease rendering them more susceptible for ischemic stroke. In line with the present study results, Wallaert et al. found a higher risk of major adverse composite events (myocardial infarction, dysrhythmia, congestive heart failure, wound infection, major amputation, and renal insufficiency) among patients with DM following lower extremity bypass surgery.^32^ Beaulieu et al. studied the risk of postoperative myocardial infarction after major vascular surgery and found a high risk of AMI following peripheral bypass surgery, with approximately 49% having DM among those suffering from AMI postoperatively.^7^ Two randomized controlled trials^33,34^ have shown that low-dose rivaroxaban taken twice a day plus aspirin once a day reduced major adverse cardiovascular and limb events when compared with ASA alone; therefore, it is of great importance to consider that patients are treated with the best medical therapy not only after the procedure but perhaps at an earlier stage.

Renal impairment is a well-known risk factor for cardiovascular morbidity and mortality among patients with DM.^35^ In accordance with previous studies, we found that renal impairment was related to a higher risk of MACE, cardiovascular mortality, and total mortality.

### Study strengths and limitations

The major strengths of the present study are the relatively long follow-up time of over 4 years, and the use of two disease-specific nationwide data registries, Swedvasc and NDR, along with data from other nationwide registries. The propensity score adjusted analysis, adjusting for approximately 30 variables, helped in minimizing the risk of confounding. The fact that only patients undergoing urgently planned open vascular surgery for infrainguinal arterial disease with CLTI were included in this study helped to lessen the risk of treatment selection bias. Furthermore, it was possible to specify the severity of CLTI, rest pain only or tissue loss, and the type of surgery performed – vein or synthetic bypass or thromboendarterectomy.

Owing to the retrospective study design, there is a potential risk of misclassification, data collection errors, and missing data leading to residual confounding. Even though the study cohort is large, it cannot be excluded that the nonsignificant association between DM and major amputation might be attributed to a type II statistical error. However, the associations between DM and the composite endpoint major amputation/mortality and all-cause mortality were weaker, which therefore favours the main interpretation of this study. Furthermore, no adjustment according to type of antidiabetic medication was done. Previous studies have shown a relation between insulin dependency and a higher risk of major amputation among patients with CLTI.^8^ Therefore, separate analyses of insulin-treated and noninsulin-treated patients would have been interesting. It should also be noted that smoking status is more fully covered in the group with DM than in the group without DM; when data on smoking status was missing in Swedvasc, complementary data were extracted from NDR. Prior studies have indicated that almost 50% of data on smoking status is missing in Swedvasc.^36^ The probably underreported level of current smoking in Swedvasc resulted nevertheless in a higher smoking rate for patients without DM compared to those with DM in the present study, which may have contributed to the comparably less unfavourable results for the DM group. Linkage of data from the prescribed drug register showed that DM and non-DM patients at baseline in the present study had rather good coverage of lipid-lowering agents and acetylsalicylic acid, but lipid-lowering therapy has improved further, as shown in the latest annual report from NDR and Swedvasc.^20,37^ The results of the present study are valid for Sweden and cannot easily be generalized to other countries.

Owing to the retrospective nature of the study, information on amputation laterality could not be retrieved. Baubeta Fridh et al. have previously reviewed the medical records of 1366 patients having major amputation due to CLTI, showing < 10% missing data for amputation with remaining uncertainty of the laterality of the amputation.^21^ Swedvasc has not yet been validated for procedures related to PAD, but Djerf et al. found that almost half of patients registered in Swedvasc due to major amputation following intermittent claudication in fact had CLTI.^38^ Therefore, the risk of misclassification of CLTI as intermittent claudication was probably low in the present study. It cannot be ruled out, however, that some patients were reclassified as having CLTI if the surgery for intermittent claudication failed.

## Conclusion

Open vascular surgery is still a first-line option in a substantial number of patients with CLTI, especially for limb salvage in patients with DM. The higher incidence rates of stroke and AMI among patients with DM following open vascular surgery for infrainguinal CLTI compared to in those without DM require specific consideration preoperatively with the aim of optimizing medical treatment in order to improve cardiovascular outcome postoperatively.

## Supplemental Material

sj-docx-1-vmj-10.1177_1358863X211008249 – Supplemental material for Diabetes mellitus was not associated with lower amputation-free survival after open revascularization for chronic limb-threatening ischemia – A nationwide propensity score adjusted analysis Click here for additional data file. Supplemental material, sj-docx-1-vmj-10.1177_1358863X211008249 for Diabetes mellitus was not associated with lower amputation-free survival after open revascularization for chronic limb-threatening ischemia – A nationwide propensity score adjusted analysis by Erika Lilja, Anders Gottsäter, Mervete Miftaraj, Jan Ekelund, Björn Eliasson, Ann-Marie Svensson, Moncef Zarrouk and Stefan Acosta in Vascular Medicine
