# Supplementary material for: Diabetes mellitus was not associated with lower amputation-free survival after open revascularization for chronic limb-threatening ischemia – A nationwide propensity score adjusted analysis
Source: Vasc Med. 2021 May 18;26(5):507–14. doi: 10.1177/1358863X211008249 (PMC8493412; doi:10.1177/1358863X211008249)
Supplement: sj-docx-1-vmj-10.1177_1358863X211008249 – Supplemental material for Diabetes mellitus was not associated with lower amputation-free survival after open revascularization for chronic limb-threatening ischemia – A nationwide propensity score adjusted analysis [file sj-docx-1-vmj-10.1177_1358863X211008249.docx]

**Appendix 1.** List of baseline variables adjusted for in the inverse probability of treatment weighting (IPTW) adjusted Cox regression.

- Age
- Sex
- Smoking
- Lipid lowering treatment
- Antihypertensive treatment
- ASA
- Clopidogrel
- Other anticoagulant therapy
- ACE
- ARB
- Alpha blocker
- Beta blocker
- Calcium channel blocker
- Diuretic
- Digoxin
- Nitrate
- Disposable income
- Education
- Civil status
- Country of origin
- AMI
- CHD
- Stroke
- AF
- HF
- Renal impairment
- Renal disease
- Cancer disease
- Liver disease
- Psychiatric disorder
- COPD
- Previous amputation
- Tissue loss
- TEA
- Vein bypass
- Synthetic or synthetic plus vein bypass
